# Migraine vestibulopathy in three families with ıdiopathic scoliosis: a case series

**DOI:** 10.1186/1757-1626-2-9367

**Published:** 2009-12-21

**Authors:** Alev Uneri, Senol Polat, Onder Aydingoz, Aysegul Bursali

**Affiliations:** 1 Department of Otorhinolaryngology, Balance Center, Acıbadem Health Group Koztayagi Hospital, Istanbul, 34742, Turkey; 2 Department of Orthopaedics and Traumatology, Cerrahpasa Medical Faculty, Istanbul University, Istanbul, 34742, Turkey; 3 Department of Orthopaedics and Traumatology, Baltalimani Bone Diseases Education and Research Hospital, Istanbul, 34742, Turkey

## Abstract

**Introduction:**

We assessed clinical and etiological association between vestibular pathology and idiopathic scoliosis concerning seven members of three families with idiopathic scoliosis.

**Case presentation:**

The families were referred to neurotology center for evaluation of balance problems. Patients were evaluated thorough anamnesis to relevant vestibular and audiological studies in addition to idiopathic scoliosis assessment. All evaluated scoliotic patients had clinical manifestations of vestibular dysfunctions and migrainous headache. All of the scoliotic patients (seven patients) in these three families were diagnosed as migraine vestibulopathy.

**Conclusion:**

With the presentation of these three families, we discussed the probable role of the vestibular dysfunctions including migraine vestibulopathy in the development and progression of idiopathic scoliosis.

## Introductıon

Idiopathic scoliosis (IS) is a three-dimensional deformity of the spine with lateral curvature combined with axial rotation. It is a permanent deviation that develops in the period of body growth, particularly during the preadolescent and adolescent period. It occurs in 2-4% of the population and affects females more than males and comprises 70% of all scoliosis cases [1].

Although the search for the etiology of IS has focused on the structural elements of the spine, spinal musculature, collagenous structures, endocrine system, central nervous system and genetics, none has shown convincing evidence of the cause. Among these studies the role of hereditary or genetic factors in the development of IS has been widely accepted [2,3].

Scoliosis may be present in various neurologic disorders involving different sites of nervous system. Especially the vestibular apparatus deserves a special attention because of its important contribution to the regulation of postural tone. Several lines of evidence indicate that adolescents affected by IS show signs of abnormal vestibular function [4,5].

In this article, as a preliminary report we tried to point out the possibility of etiological and clinical relationship between IS and vestibular disorders in three families with scoliosis that were treated for the migraine vestibulopathy.

## Case presentatıons

In this study we reported three families consisted of seven white patients belonging to Turkish nationality which were referred to Acibadem Health Group Kozyatagi Hospital Otorhinolaryngology, Neurotology Clinic between September 2006-april 2007. The study protocol was approved by the ethical board of the hospital. All of the members of these families were evaluated thorough anamnesis including the key features of their complaints, together with general physical and neurotologic examination, in addition to relevant vestibular and audiological studies.

Audiological tests included pure tone audiometry, impedans audiometry and speech discrimination. Vestibular studies comprised of balance tests, videonystagmography (VNG), electronystagmography (ENG) and sensory organization tests (SOT) with computerized dynamic posturography (CDP). For the definition of migraine vestibulopathy, we used the diagnostic criteria that is described in Appendix 1. which we have been using since January 2000 [6].

### Case Report 1

Three members of a family that had been treated for IS were referred to the neurotology clinic because of dizziness and vertigo attacks. Radiographs showed idiopathic curves ranging in severity from 30 degrees in the youngest child examined, a five-year-old girl, to 17 degrees in the boy of thirteen. The mother had right thoracic curve about 14 degrees. On neurotological evaluation; The mother had a history of imbalance and dizzy spells for about twenty years with intermittent unilateral pounding head pain associated with phonophobia and occasionally photophobia. In Electronystagmographic tests: There was a spontan nystagmus, only visible when eyes are closed, and whose maximal slow phase velocity was 12°/s right. Additionally, on Hallpke examination, this nystagmus was continuing in both sides, without changing its slow phase and plane, only changing its velocity; 17°/s on the right side and 5°/s on the left side. Optokinetic, Saccad and Pursuit tests were in normal range. Provocative movement of the head in any direction resulted in severe vertigo and nausea. Hearing was normal except mild sensorineural decrease in low frequencies.

Daughter: This patient initially presented at age 5 with episodes of headaches, often preceded by an aura described as rows of sparkling dots blocking her vision and persisting for approximately 15 minutes. The mother reported that her daughter had abrupt spontaneous attacks of vertigo and dizzy spells each spontaneously remitting after several days. On ENG examination bilateral caloric tests were symetrical. Dix-Hallpike positioning tests revealed left beating nystagmus with a slow phase velocity 10°/s. which was triggered by moving the patient from the sitting to head-hanging both left and right positions.

Son: This patient presented at age 13 and described episodes of vertiginous attacks and imbalance beginning approximately at age 9. Migrainous headaches with occasional visual aura began in his early childhood, persisting up to the present time. On VNG and ENG examination right beating spontaneous nystagmus was detected and caloric tests revealed hypoactivity on the right side, with a %30 assymetry. Audiometric tests were normal.

### Case Report 2

Two sisters with IS were presented with episodic attacks of dizziness and vertigo. Radiographs showed idiopathic curve of 20 degrees in the older woman, 58-year-old, and moderate curve about 15 degrees in the younger sister, 54-year-old.

Sister 1: This patient, the younger sister, presented with episodic vertiginous attacks dating back approximately 3 years. She reported several similar bouts of attacks dating back to teenage years. Approximately at age 20, she began having unilateral pulsating headaches associated with photophobia, phonophobia, nausea and occasionally vomiting. Videonystagmographic examination revealed right nystagmus that worse upon right gaze and Dix-Hallpike maneuver accelerated nystagmus without a rotational component. Bilaretal caloric tests were hypoactive with the responses less than 10°/s and she had flat type moderate sensorineural hearing loss in both ears.

Sister 2: This patient, the older sister, had complaint of chronic dizziness and headlightliness each lasting 1 to 6 months and occasional vertiginous attacks throughout her adolescent and adult life. She experienced dull head pains in her childhood, and during adolescence she began having unilateral headache attacks with phonophobia. On VNG examination right beating spontaneous nystagmus was detected. Caloric tests revealed hypoactivity on the right side. with a %27 assymetry. She had moderate level sensorineural hearing loss in low frequencies on the right ear.

### Case Report 3

Thirty years old woman and her mother, 59-year old, both with IS were presented with episodic attacks of dizziness and vertigo. Radiographs showed idiopathic right lomber curve of 22 degrees in the mother, and right lomber curve about 15 degrees in the doughter.

Daughter: This patient initially presented with vertigo and dizziness 3 months ago. She stated dizzy attacks and vertigo that were needed hospitalization for two times in her younger life. She described episodes of headaches, often preceded by photophobia persisting for approximately 15 minutes. In Electronystagmographic tests: There was a spontan nystagmus and whose maximal slow phase velocity was 14°/s right. She had right beating nystagmus in right, and left beating nystagmus in left side in Dix-Hallpike maneuver with VNG examination.

Mother: This patient was presented with left sided hearing loss, tinnitus in the same side, dizziness and vertigo. Hearing in the left ear was gradually decreased. She described intermittent unilateral headache associated with phonophobia and occasionally photophobia. Videonystagmographic examination and ENG Tests revealed right sided spontaneous nystagmus and slow phase velocity was 17°/s. She had flat type severe sensorineural hearing loss (more than 70 dB) in the left ear.

In these all cases radiological studies revealed no central nervous system pathology. On CDP examination SOT scores were normal in all patients.

## Dıscussıon

We presented three families with IS that were diagnosed as migraine vestibulopathy. We could not find this kind of presentation in English literature. In these cases the clinical presentation of the headaches fulfilled the criteria of the International Headache Society. Patients had vestibular complaints, such as dizzy spells, vertiginous attacks and intermittent balance problems. Neurotologic examinations revealed vestibular signs, such as nystagmus with VNG or ENG testing.

The clinico-epidemiological association between migraine and vestibular signs and symptoms suggests that they may have common mechanisms. The connections between the vestibular nuclei, the trigeminal system and the thalamocortical nuclei offer a rationale for the development of patho-physiological model of migraine vestibulopathy. Over the last few decades clinico-epidemiological and causal relationship between vertigo and migraine has evolved from several patient-control series [7]. The mere association of vertigo and migraine is certainly insufficient to make the diagnosis of migraine vestibulopathy, as vestibular disorders and migraine have both a high prevalence in the general population. So various sets of criteria have been proposed [7,8] with a common prerogative to all criteria is the exclusion of other causes of vertigo. In this study, we used the diagnostic criteria that had been established by senior author in the definition of this vestibular disturbance.

Vestibular apparatus is important in regulation of postural and oculomotor control. Postural righting reactions depend on the integration of information from the visual system, the somatosensory system, and integrative vestibular system with vestibulo-spinal, vestibulocerebellar and the vestibulo-ocular pathways. When the eyes closed, righting with gravity is more dependent on the sensory system and the vestibular system[9]. It was suggested that vestibular information is used as a gravitational reference frame to prevent slow drift of the trunk in space during complex postural tasks[10], and it was postulated that scoliotic patients were particularly sensitive to dynamic perturbations of posture and they might fail complex balance tasks that challange the vestibular system [9,1,1]. In addition to the postural asymmetry, the presence of spontaneous and positional nystagmus observed among IS patients may also indicate the presence of an asymmetric vestibular function [12]. Four of our patients had spontaneous and others had positional nystagmus.

In addition to these arguements, it is known that vestibular afferents may influence the activity of parietal and temporal cortical regions, and thalamic nuclei, where they interact with visual and proprioceptive volleys. Therefore, Manzoni proposes that vestibular dysfunction may affect this integrative process leading to abnormal motor commands which may establish a spinal deformity. Also, vestibular signals could regulate the activity of the spinal and cerebellar circuits which are involved in oculomotor and postural control and disruption of the normal vestibular activity may triger the development of scoliosis [2].

There are many studies reporting genetic aspect of the IS that is known to aggregate within families, and multiple clinical studies support either an autosomal dominant, multifactorial, or X-linked inheritance pattern for IS [3,12]. Ogilvie JW et al. studied a cohort of 145 patients with adolescent idiopathic scoliosis(AIS) and founded that nearly all (97%) AIS patients have familial origins [13]. Thus, while the specific cause of adolescent idiopathic scoliosis has not been established, the role of hereditary or genetic factors in the development of this condition is widely accepted. In one study, it has been suggested that an autosomal gene factor may create a mild central nervous system abnormality that predisposes an adolescent to IS, also it was proposed that, problems with vestibular imbalance could increase the risk for developing progressive IS in individuals with an autosomal recessive gene for scoliosis [14]. In deed, it is clear that all individuals with vestibular imbalance do not develop IS and all of the patients with IS do not have vestibular dysfunctions. Hence, in a subgroup of patients with IS, the spinal deformity and the vestibular disorders could develop together, under the pressure of a common etiological factor which might be genetically coded, moreover, a vestibular imbalance could be the consequence of a spinal deformity.

## Conclusıon

With the presentation of these three families, we tried to point out the probable association between the vestibular pathology and IS. The clinical implication is that; Vestibular system evaluations should be included in the assessment of the etiological and clinical studies concerning IS. Also even if the minority of the scoliotic patients might have chronic vestibular dysfunction, it is possible that integrative vestibular treatment and rehabilitation could increase postural righting reactions and could decrease the risk for curve progression.

## Competing interests

The authors declare that they have no competing interests.

## Authors' contributions

OA and AB played role in diagnosis and treatment the scoliotic manifesrations, they helped in collecting and presenting the demograhic data of the patients. AU and SP played role in diagnosis and treatment the neuro-otologic menifestations. They helped in collecting data, analaysis and writting the manuscript. All authors read and approved the final manuscript.

## Consent

Written informed consent were obtained from the patients and from their parents for publication of this case report. Copies of the written consents are available for review by the Editor-in-Chief of this journal.

## Appendix 1 - Criteria for Migraine vestibulopathy

### Group A criteria

1. Dizziness

a. Chronically on-going dizziness (4 weeks up to several years)

b. Episodic attacks of dizziness (in between few seconds to days)

c. Dizziness continuous after vertigo attacks (more than one day)

2. Vertigo attacks

a. Vertigo attacks with short durations (brief periods) (from few seconds to 15 minutes)

b. Classical vestibular attack (from 15 minutes to 72 hours)

### Group B criteria

1. To fit at least one of the migraine definition in past history, according to International Headache Society classification (lifetime diagnosis of migraine).

2. Migraine presence in first degree relatives Motion Sickness (especially past history of childhood period) Low blood pressure; (casual SBP of < 105 mm hg and/or DBP of < 60 mm Hg)

The patients termed as migraine vestibulopathy; if they had got at least one of the criteria from Group A with criterion 1 from Group B, or with at least two criteria in subclasses 2 of Group B, afer exclusion of other causes of vertigo.

## References

[B1] Byl NN Holland S Jurek A Hu SS Postural imbalance and vibratory sensitivity in patients with idiopathic scoliosis: implications for treatment J Orthop Sports Phys Ther 1997 26 2 60 68 9243403 10.2519/jospt.1997.26.2.60

[B2] Manzoni D Miele F Vestibular mechanisms involved in idiopathic scoliosis Arch Ital Biol 2002 140 1 67 80 11889923

[B3] Giampietro PF Blank RD Raggio CL Merchant S Jacobsen FS Faciszewski T Shukla SK Greenlee AR Reynolds C Schowalter DB Congenital and idiopathic scoliosis: clinical and genetic aspects Clin Med Res 2003 1 2 125 136 10.3121/cmr.1.2.125 15931299 PMC1069035

[B4] Wiener-Vacher SR Mazda K Asymmetric otolith vestibulo-ocular responses in children with idiopathic scoliosis J Pediatr 1998 132 6 1028 1032 10.1016/S0022-3476(98)70403-2 9627598

[B5] Sahlstrand T Petruson B Ortengren R Vestibulospinal reflex activity in patients with adolescent idiopathic scoliosis. Postural effects during caloric labyrinthine stimulation recorded by stabilometry Acta Orthop Scand 1979 50 3 275 281 314221 10.3109/17453677908989768

[B6] Uneri A Polat S Vertigo, dizziness and imbalance in the elderly J Laryngol Otol 2008 122 5 466 469 10.1017/S0022215107000424 17850686

[B7] Cass SP Furman JM Ankerstjerne K Balaban C Yetiser S Aydogan B Migraine-related vestibulopathy Ann Otol Rhinol Laryngol 1997 106 3 182 189 9078929 10.1177/000348949710600302

[B8] Crevits L Bosman T Migraine-related vertigo: towards a distinctive entity Clin Neurol Neurosurg 2005 107 2 82 87 10.1016/j.clineuro.2004.07.003 15708220

[B9] Byl NN Holland S Jurek A Hu SS Postural imbalance and vibratory sensitivity in patients with idiopathic scoliosis: implications for treatment J Orthop Sports Phys Ther 1997 26 2 60 68 9243403 10.2519/jospt.1997.26.2.60

[B10] Horak FB Jones-Rycewicz C Black FO Shumway-Cook A Effects of vestibular rehabilitation on dizziness and imbalance Otolaryngol Head Neck Surg 1992 106 2 175 180 1738550

[B11] Byl NN Gray JM Complex balance reactions in different sensory conditions: adolescents with and without idiopathic scoliosis J Orthop Res 1993 11 2 215 227 10.1002/jor.1100110209 8483034

[B12] Sahlstrand T Petruson B A study of labyrinthine function in patients with adolescent idiopathic scoliosis. I. An electro-nystagmographic study Acta Orthop Scand 1979 50 6 Pt 2 759 769 534551 10.3109/17453677908991307

[B13] Ogilvie JW Braun J Argyle V Nelson L Meade M Ward K The search for idiopathic scoliosis genes Spine 2006 31 6 679 681 10.1097/01.brs.0000202527.25356.90 16540873

[B14] Bell M Teebi AS Autosomal dominant idiopathic scoliosis? Am J Med Genet 1995 55 1 112 10.1002/ajmg.1320550126 7702081

